# Unravelling the pathological roles of anastasis in cancer recurrence

**DOI:** 10.1098/rsob.240270

**Published:** 2025-06-25

**Authors:** Ho Man Tang, Ho Lam Tang

**Affiliations:** ^1^ Department of Neurosurgery, Johns Hopkins Medicine, Baltimore, MD, USA

**Keywords:** anastasis, apoptosis, cancer recurrence, metastasis, mutagenesis, reversal of apoptosis

## Abstract

Can anastasis contribute to cancer recurrence? Anastasis is a cell recovery mechanism to spare dying cells after the initiation of the cell death process. Emerging studies interrogate anastasis as an unexpected escape tactic for cancer cells to evade cell death-inducing anti-cancer therapy, leading to recurrence. After anastasis, cancer cells display increased invasiveness and genomic instability, which could be associated with the common and fatal features of metastasis and drug resistance at the cancer recurrence. These studies open an encouraging new conceptual avenue for arresting cancer recurrence by targeting anastasis in cancer cells after conventional anti-cancer therapy. Here, we highlight recent findings towards unravelling pathological roles of anastasis in cancer recurrence, for the purpose of stimulating ideas and promoting the development of this new field of cancer research.

**Figure:**
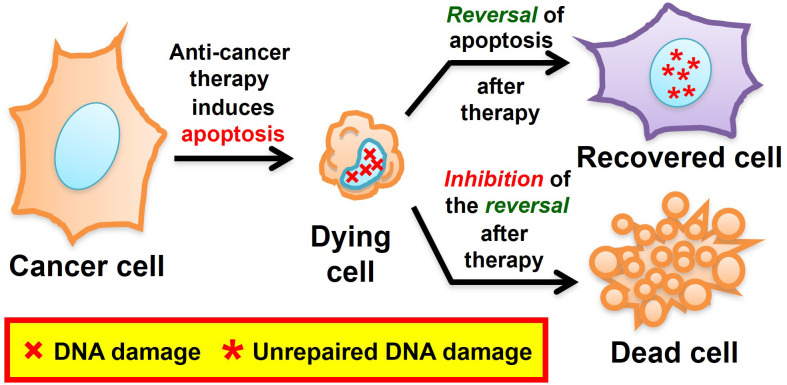

## 1. Introduction: reversing apoptosis by anastasis—a novel therapeutic target

**Figure 1.**
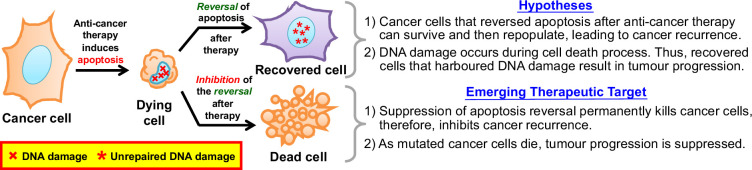
Therapeutic potential of anastasis in cancer treatment. The discovery of anastasis in cancer cells reveals a cytoprotective mechanism that dying cancer cells could use to escape cell death-inducing anti-cancer therapy by reversing apoptosis and harbour DNA damage generated by the mechanisms of apoptosis and the genotoxic therapy, leading to cancer recurrence and progression. Therefore, targeting anastasis is a potential therapeutic strategy to arrest cancer recurrence by inhibiting the recovery of dying cancer cells, leading to a cure.

Cancer recurrence is a major problem in the clinical management of most types of cancer [1,2]. Activating **apoptosis** (Greek for ‘falling to death’ [3]; see Glossary, table 1), a form of **programmed cell death** [4,5], is an important therapeutic strategy that eliminates cancer cells by prompting them to die through cell suicide [6]. Apoptosis-inducing therapies can cause cancer cell death and tumour shrinkage. However, most cancers recur, leading to treatment failure [6]. The causes of cancer recurrence are diverse and complex, such as solid tumours impenetrable to cancer drugs, heterogeneous tumour cell responses, and tumour stem cell self-renewal [2,7]. The recent discovery of **anastasis** (Greek for ‘rising to life’, referring to ‘**reversal of apoptosis**’ here; Box 1) in cancer cells reveals an emerging therapeutic target for arresting cancer recurrence after conventional apoptosis-inducing therapies [8–14]. Please see figure 1 for reference throughout.

Box 1. **‘Reversing apoptosis’ versus ‘resisting apoptosis’.**

‘Reversing apoptosis’ refers to anastasis, in which dying cells can recover from an already initiated apoptotic cell death process. This is different from ‘resisting apoptosis’, in which no apoptosis has been initiated. For example, in response to a death stimulus, anastatic cells are those that first undergo apoptotic events (e.g. mitochondrial outer membrane permeabilization, caspase-3 activation, DNA damage) and then recover from these cellular crises and continue to survive. In contrast, the cells that resist apoptosis do not respond to the death stimulus as no apoptotic event was triggered.

### 1.1. Prevailing assumptions of irreversible apoptosis

**Figure 2.**
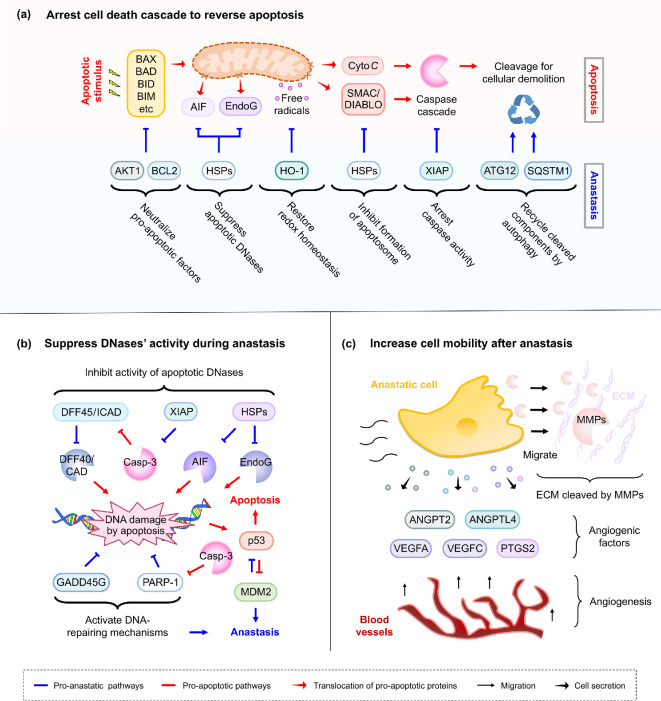
Emerging mechanisms of anastasis. (a) Pro-anastatic pathways interact with pro-apoptotic pathways to arrest the cell death cascade for rescuing dying cells by reversing apoptosis. (b) Anastasis suppresses the activity of apoptotic DNases during reversal of apoptosis and inhibits p53-mediated cell death-inducing DNA damage response. (c) Anastatic cells upregulate MMPs and angiogenic factors. Abbreviations: AIF, apoptosis-inducing factor; AKT1, Akt serine/threonine kinase 1; ANGPT2, angiopoietin 2; ANGPTL4, angiopoietin-like 4; ATG12, autophagy-related 12; BAD, BCL2 associated agonist of cell death; BAX, BCL2 associated X; BCL2, B-cell lymphoma 2; BID, BH3 interacting-domain death agonist; BIM, BCL2 interacting mediator of cell death/ BCL2-like protein 11; Casp-3, caspase-3; Cyto *C*, cytochrome *c*; DFF40/CAD, DNA fragmentation factor 40/caspase-activated DNase; DFF45/ICAD, DNA fragmentation factor 45/inhibitor of caspase-activated DNase; ECM, extracellular matrix; EndoG, endonuclease G; GADD45G, growth arrest and DNA-damage inducible 45 gamma; HO-1, heme oxygenase; HSPs, heat shock proteins; MDM2, mouse double minute 2; MMPs, matrix metalloproteinases; PARP-1, poly(ADP-ribose) polymerase 1; PTGS2, prostaglandin-endoperoxide synthase 2; SMAC/DIABLO, second mitochondria-derived activator of caspases/direct inhibitor of apoptosis-binding protein with low pI; SQSTM1, sequestosome 1; VEGFA, vascular endothelial growth factor A; VEGFC, vascular endothelial growth factor C; XIAP, X-linked inhibitor of apoptosis protein.

Apoptosis has long been assumed to be an intrinsically one-way process ending in cell suicide because it is both rapid and massively destructive [15,16]. Upon apoptosis initiation, pro-apoptotic factors (e.g. BAX, BAD, BID, BIM) translocate to mitochondria to trigger **mitochondrial outer membrane permeabilization** (MOMP) [17,18]. Once MOMP causes mitochondria to release apoptogenic factors (e.g. cytochrome *c* and SMAC/DIABLO) into the cytosol, apoptotic proteases called **caspases** can be activated [4,19], followed by cytoplasmic and nuclear condensation within a few minutes and cell death shortly thereafter [4] (figure 2*a*). Considering that dysfunctional energy production of damaged mitochondria and initiation of the proteolytic caspase cascade for cellular demolition can each independently cause cell death, recovery from such enormous destruction seems intuitively unlikely [19–21].

### 1.2. Reversal of late-stage apoptosis in cancer cells

Challenging the prevailing view of irreversible apoptosis, compelling evidence reveals that dying cancer cells can recover from a stage previously thought incompatible with life. Time-lapse live-cell microscopy has demonstrated that even after the generally accepted ‘**point of no return**’ (e.g. cytochrome *c* release, caspase-3 activation, and subsequent cellular damages, including nuclear condensation, DNA damage, cell shrinkage and plasma membrane blebbing), **dying cells** can regain normal morphology and cellular functions, and then continue to survive and divide *in vitro* [8–14]. Dying cancer cells can reverse apoptosis resulting from generic cell death inducers (e.g. ethanol, staurosporine, dimethyl sulfoxide) or anti-cancer agents (e.g. adriamycin, cisplatin, paclitaxel; discussed below). These findings established the reversibility of apoptosis even after critical steps once deemed irreversible in cancer cells.

### 1.3. Developing concept of anastasis in cancer recurrence

Anastasis could be a general cytoprotective mechanism that enables apoptotic dying cancer cells to survive apoptotic cell death-inducing anti-cancer therapy, causing cancer recurrence. Simply removing cell death inducers is sufficient to allow dying cancer cells to reverse apoptosis [8–14]. Many chemotherapy and radiation therapy induce apoptosis and are delivered episodically with recovery periods between successive treatments [6,22]. This approach is similar to applying an apoptosis inducer and then washing it away, thereby allowing apoptotic cancer cells to undergo anastasis and cancer recurrence. Therefore, inhibiting anastasis in apoptotic cancer cells would allow them to die, thereby preventing the recurrence of the cancer, leading to a cure.

## 2. Pathological potential of anastasis in cancer recurrence

Genomic instability [2] and metastasis [23] are common and often fatal features of cancer recurrence that could be associated with anastasis in cancer cells (figure 2*b*,*c*).

### 2.1. Genomic instability

Anastasis could promote **mutagenesis** in cancer cells. DNA damage is the hallmark of apoptosis [4]. In dying cells, DNA is cleaved by **apoptotic DNases**, such as MOMP-released apoptosis-inducing factor (AIF) and endonuclease G (EndoG), and caspase-activated DNA fragmentation factor/caspase-activated DNase (DFF40/CAD), whereas DNA repair enzymes such as PARP-1 are cleaved and destroyed by activated caspase-3 [4,17]. Therefore, anastatic cells could harbour and perpetuate mutations from DNA damage generated during apoptosis (figure 2*b*). This notion is supported by increased genetic alterations in anastatic cancer and non-cancerous cells, such as in ethanol- or BH3-mimetic-treated human cervical and bone cancer cells, as well as in ethanol-treated mouse primary liver cells, dimethyl sulfoxide-treated mouse embryonic fibroblasts, and radiated human mammary epithelial cells, that survived after they had experienced transient activation of cell death mechanisms, as shown in time-lapse live-cell imaging, karyotyping, micronucleus, or transformation assays *in vitro* [8,24–26].

Genetic alterations in anastatic cells could underline the observation that recurrent tumours often display drug resistance after repeated rounds of cancer treatment [2,27]. If anastasis happens in cancer cells between cycles of genotoxic anti-cancer therapy, DNA-damaged cancer cells surviving by anastasis could acquire new mutations, driving cancer evolution and drug resistance. Also of concern, anti-cancer therapy can trigger apoptosis in drug-sensitive normal cells, such as haematopoietic stem cells in bone marrow, due to off-target effects [6]. If anastasis occurs in the stem cells with DNA damage caused during apoptosis, leukaemogenesis could occur, possibly accounting for the appearance of second (new) cancers, such as acute myeloid leukaemia, even after the completion of cancer treatment that destroyed the first (primary) tumours. Reversing apoptosis in DNA-damaged cells may also contribute to carcinogenesis when normal tissues are exposed to repeated cell death-inducing stresses and injuries, such as association of liver cancer to chronic alcohol consumption [28,29], and oesophageal cancer to hot food and beverage consumption [30,31].

### 2.2. Metastasis

Anastasis could promote cancer cells to migrate away from their primary tumour sites after anti-cancer therapy, leading to **metastasis**. This notion is supported by time-lapse live-cell imaging, wound-healing assays and transwell assays that demonstrated an increased mobility and invasiveness of adriamycin- or cisplatin-treated breast cancer cells [32], paclitaxel-treated colorectal and ovarian cancer cells [33,34], ethanol-treated cervical cancer cells [12,13], and doxycycline-mediated tBID-induced skin cancer cells [35], after these human cancer cells had recovered *in vitro* from apoptotic induction, as compared with corresponding untreated cells. Grafting cells recovered from apoptosis in animals demonstrated the increased malignancy *in vivo* for adriamycin- or staurosporine-treated breast cancer cells [32,36], paclitaxel-treated ovarian cancer cells [33] and BH3 mimetics-treated lung cancer cells [37] in mice, as well as the tBID-induced skin cancer cells in mice, zebrafish embryos, and embryonated chicken eggs [35].

Mechanistically, anastatic cervical, liver, and ovarian cancer cells upregulate genes involved in cell migration (**matrix metalloproteinases**: MMP9, MMP10, MMP13) and angiogenesis (**pro-angiogenic factors**: ANGPT2, ANGPTL4, VEGFA, VEGFC, PTGS2) [12,13,33]. Anastatic breast cancer cells also upregulate the **cell adhesion** protein cadherin 12 (CDH12) [32] *in vitro*. These findings suggest a mechanistic connection between anastasis and cancer metastasis during recurrence: an increased mobility for anastatic cancer cells could enable them to escape the adverse microenvironment in tumours resulting from cell death after anti-cancer treatment. Additionally, the expression of angiogenic factors, which promote angiogenesis and vascular permeability, could promote the progression of anastasis by delivering nutrients to recovering cells. These factors could also facilitate cell migration, promoting metastasis, new tumour formation, and cancer recurrence (figure 2*c*).

## 3. Emerging mechanisms of anastasis

Whole-genome gene expression, proteomics, and RNAi screening studies have begun to uncover molecular mechanisms and key regulators governing reversal of apoptosis (figure 2) [12,13,33,34,38–40]. When reversing apoptosis, anastatic cells adopt varied strategies to arrest cell death cascade and restore cellular damage. Although the molecular signature of anastasis can vary between cell types and death stimuli, overall the emerging anastatic mechanisms appear to share increased expression of the following: XIAP to arrest caspase-mediated destruction, AKT1 and pro-survival BCL2 family members to suppress MOMP-mediated apoptotic signals, MDM2 to suppress p53-mediated death signalling associated with the DNA damage response pathway; DFF45/ICAD and HSPs to arrest apoptotic DNases, PARP-1 and GADD45G to repair DNA damage, HO-1 to neutralize free radicals generated in apoptosis, ATG12 and SQSTM1 to remove damaged cellular components by autophagy, angiogenic factors to promote growth of blood vessels towards anastatic cells for nutrient uptake, and AP-1 to coordinate transcriptions for cell repair [12,13,33,34,38,39] (figure 2*a*). These mechanisms are potential therapeutic targets for controlling anastasis.

## 4. Open questions

The ‘point of no return’ of the cell death process is still unclear. The final destiny of **dead cells** is to be cleared in the body [41]. For cell death mediated by apoptosis, dying cells display ‘eat me’ signals such as phosphatidylserine (PS) on the cell surface, leading to engulfment and clearance of apoptotic cells, such as by **efferocytosis**, in which they are broken down and digested [42,43]. Whether engulfed dying cells can escape from this clearance mechanism is an open question. Interestingly, studies revealed that the PS-exposed dying cells can recover from apoptosis and survive [8,44–46]. Moreover, live internalized cells undergoing a non-apoptotic cell death process called **entosis** can have a chance to be released and stay alive [47,48]. Therefore, it is possible that checkpoints once thought to be inevitable to cell death, such as being engulfed, and also releasing cytochrome *c* and activating caspases as discussed above, can be managed by dying cells to recover from the brink of cell death by undergoing anastasis. Defining the point of no return, which could vary between cell types and cell conditions, will promote our understanding of when, where, and how anastasis occurs, and will reveal the druggable targets of anastasis for therapeutic intervention.

## 5. Concluding remarks

The ability of anastasis to reverse apoptosis in cancer cells with metastatic and mutagenic properties offers new challenges and opportunities for anti-cancer therapy. To harness the discovery of anastasis for developing new therapies, the first crucial step is to identify the master regulators of anastasis, which are potentially druggable targets. While multiple mechanisms for reversing apoptosis have been proposed, definitive upstream regulators orchestrating such mechanisms have remained unclear. In addition to providing leads for developing a new class of anti-cancer agents, identifying these master regulators will generate valuable new knowledge for future pathophysiological and mechanistic studies of anastasis. Manipulating anastasis could significantly improve the outcomes of cell death-inducing cancer therapies.

**Table 1.** Glossary.

Glossary	
Anastasis	Anastasis (*αναστάσης*, a Greek word for ‘rising to life’) is a natural cell recovery mechanism that rescues dying cells before the completion of the cell death process [8]. Reversal of apoptosis is an example of anastasis, which has been demonstrated in cancer and non-cancerous cells *in vitro* [9], and also in somatic and germ cells in fruit flies *in vivo* [49–51].
Apoptosis	Apoptosis (Greek for ‘falling of leaves to death’) is a cell suicide process that plays an essential role in eliminating unwanted, damaged, or otherwise dangerous cells in multicellular organisms [3,52]. Defective apoptosis can lead to intractable diseases such as cancers [2,6].
Apoptotic DNases	DNases that cleave DNA during apoptosis. Different apoptotic DNases are activated by distinct apoptotic events, such as (i) MOMP to release AIF and EndoG from mitochondria to cytosol, and (ii) caspase activation to cleave DFF45/ICAD to remove its inhibition to DFF40/CAD, before they can cleave the genome in an apoptotic cell [17].
Caspases	Family of proteolytic enzymes that serve as key executioners in apoptosis by mediating cellular demolition through cleaving hundreds of proteins for demise. These cysteine proteases cleave their protein substrates after aspartic acid residues. Caspase-3 is one of the effector caspases in the family and the major executioner in caspase cascade for cellular demolition [4,19].
Cell adhesion	Process for a cell to attach to its neighbour cells or extracellular matrix. Cell–cell adhesions are important to generate mechanical force for cell migration and invasion processes of metastasis [53].
Dead cells	Cells that have completed the cell death process and concluded their demise. The dead cells cannot reverse apoptosis by anastasis as they are inviable [9].
Dying cells	Cells that are undergoing the process of cell death but have not completed it. While dying cells display hallmarks of the cell death process distinguishing them from healthy cells, they are still viable and proceeding the death process and therefore differ from dead cells [9].
Efferocytosis	Engulfment of dying and dead cells by macrophages and phagocytes. The clearance of dead cells and cellular debris is essential for maintaining embryonic development and normal homeostasis in multicellular organisms. Failed efferocytosis is associated with cancer and autoimmune diseases [42,43].
Entosis	A non-apoptotic cell death process that a live cell invades into another live cell, leading to cell internalization. The live internalized cells are either degraded (to die) or released (to survive) [47,48].
Matrix metalloproteinases (MMPs)	Family of proteases that play an important role in cell migration by cleaving extracellular matrix [54,55].
Metastasis	Process for cancer cells to spread from the original tumour where they first formed to other locations to form new tumours in the body [23,56].
Mitochondrial outer membrane permeabilization (MOMP)	Critical step of apoptosis in which mitochondrial translocation of pro-apoptotic BCL-2 proteins (e.g. BAX, BAD, BID, BIM) generate pores across the outer membrane of mitochondria to release apoptogenic factors (e.g. cytochrome *c*, SMAC/DIABLO, AIF, EndoG) into the cytosol. This causes dysfunction of mitochondria (e.g. respiration/ATP production) and triggers apoptotic cascades (e.g. caspase activation, DNA damage), either of which alone can lead to cell death [17,18].
Mutagenesis	Process of causing alteration in DNA sequence, leading to a mutation. The change of genetic material in a cell can promote carcinogenesis, cancer progression, and development of drug resistance [57–59].
Point of no return	Checkpoint in a cell death process where dying cells become irreversibly committed to demise. A dying cell cannot recover through anastasis when it has passed the actual ‘point of no return’ in the cell death process. The concept of ‘point of no return’ has yet to be specifically defined. MOMP and activation of caspases have long been generally considered as the ‘point of no return’ [19–21], but recent studies show that some dying cells can reverse apoptosis and recover from these stages of cell death process [9].
Programmed cell death	Type of cell death that is controlled by a series of intrinsic ‘biological programme’ mechanisms, leading to death of the cell. Apoptosis is the first identified and best-characterized form of programmed cell death [5,60].
Pro-angiogenic factors	Biological molecules that promote angiogenesis, leading to formation of vascular networks radiating from the source of these molecules. Upregulation of angiogenesis in tumour tissues can promote metastasis [2].
Reversal of apoptosis	The cell recovery after initiation of apoptosis, bypassing the hallmark checkpoints of this cell death process, such as MOMP, caspase-3 activation, and DNA damage [9].

## Data Availability

This article has no additional data.
